# Emergence of norovirus GII.17[P16] in adult patients with acute gastroenteritis in Thailand during 2021−2023

**DOI:** 10.1371/journal.pone.0337513

**Published:** 2025-11-24

**Authors:** Leera Kittigul, Thongthiw Pairoh, Kitwadee Rupprom, Yuwanda Thongpanich, Sukhontha Siri

**Affiliations:** 1 Department of Microbiology, Faculty of Public Health, Mahidol University, Bangkok, Thailand; 2 Department of Clinical Pathology, Faculty of Medicine Vajira Hospital, Navamindradhiraj University, Bangkok, Thailand; 3 Department of Epidemiology, Faculty of Public Health, Mahidol University, Bangkok, Thailand; CEA, FRANCE

## Abstract

**Background:**

Human norovirus is a major cause of acute gastroenteritis across all age groups. This study investigated the prevalence, genotypes, and viral loads of noroviruses in adult patients with acute gastroenteritis.

**Methods:**

A hospital-based cross-sectional study was conducted between December 2021 and March 2023 in Thailand. The presence of noroviruses GI and GII in stool samples from patients with acute gastroenteritis were tested using RT-nested PCR and DNA sequencing. All norovirus GII-positive samples were further characterized by polymerase-capsid typing assay and semi-nested RT-PCR. Norovirus GII concentrations were determined by RT-qPCR.

**Results:**

Norovirus GII was detected in 11.2% (28/250) of stool samples. Genotyping of the VP1 and RdRp genes identified GII.4 Sydney 2012[P31], GII.17[P17], and GII.17[P16], with GII.17[P16] being the most frequently detected. Of 28 norovirus GII-positive samples, the most common genotype was GII.17 (35.7%), followed by GII.17[P16] (32.1%), GII.17[P17] (14.3%), GII.4 Den Haag (7.1%), and GII.4 Sydney 2012[P31], GII.3 and GII.2 (3.6% each). GII.4 Sydney[P31] infections had the highest viral load (8.3 × 10^9^ genome copies/g), followed by GII.17[P17] (8.9 × 10^5 ^− 4.5 × 10^8^ genome copies/g) and GII.17[P16] (3.7 × 10^4 ^− 1.6 × 10^7^ genome copies/g). GII.3 (2.4 × 10^4^ genome copies/g), GII.4 Den Haag (1.7 × 10⁴ and 3.8 × 10⁴ genome copies/g) and GII.17 (2.4 × 10³–7.4 × 10⁴ genome copies/g) exhibited lower viral concentrations.

**Conclusions:**

These findings provide important epidemiological insights into norovirus circulation, highlighting the emergence of GII.17[P16] and informing future outbreak preparedness and vaccine development.

## Introduction

Human norovirus is a leading cause of acute gastroenteritis, affecting individuals across all age groups. Globally, it is responsible for approximately 677 million cases of gastrointestinal illness and 213,000 deaths each year [1]. In 2019, children under 5 years old accounted for an estimated 33% of norovirus-related deaths and 56% of disability-adjusted life-years (DALYs), while adults over 70 years old represented 40% of deaths and 11% of DALYs [2]. Norovirus is a non-enveloped virus from the *Caliciviridae* family, with a positive-sense, single‐stranded RNA genome of approximately 7.7 kb. There are ten recognized genogroups, of which genogroups I (GI), II (GII), IV (GIV), VIII (GVIII), and IX (GIX) infect humans. Within these genogroups, noroviruses are further classified into at least 49 genotypes based on the VP1 capsid protein and 60 P-types based on RNA-dependent RNA polymerase (RdRp) sequences [3]. Norovirus exhibits high genetic diversity, with multiple genogroups and genotypes cocirculating in human populations. Among these, GII.4 variants consistently driven global epidemics, and GII.4 Sydney 2012 being the predominant genotype since its emergence in 2012 [4]. Other genotypes have periodically dominated outbreaks, such as GII.17[P17] genotype [5,6], along with recombinant strains like GII.4 Sydney [P31], GII.4 Sydney[P16], and GII.2[P16] [7,8].

Norovirus GII.17 strains have been circulating at low detection rates in patients with acute gastroenteritis alongside the predominant GII.4 viruses. Notably, the GII.17[P17] strains Kawasaki308 and Kawasaki323 emerged and spread globally during 2014−2015 [9,10]. Between 2016 and 2022, an increasing prevalence of recombinant strains—GII.4 Sydney[P31], GII.4 Sydney[P16], and GII.2[P16]— were reported across various countries [7,8,11]. From 2021 to 2024, the number of GII.4 infections gradually declined, while GII.17 emerged as the predominant genotype worldwide. The GII.17[P17] strain was identified in multiple regions, including United States, England, Germany, France, and Austria [6], Spain [12], Argentina [13], China [14], Romania [15], and Russia [16].

In Thailand, during 2017−2018 diverse norovirus genotypes were detected, with GII.4[P31] being predominant in sporadic cases [17]. A shift from a high prevalence of GII.2[P16] in 2017 to increased detections of GII.4[P16] and GII.4[P31] in 2018 was observed [18]. Between 2019 and 2020, GII.4 Sydney remained the predominant genotype among pediatric gastroenteritis cases [19]. From 2020 to 2022, GII.17 was the most frequently detected genotype in environmental water samples [20]. In 2023, a GII.17 strain closely related to the Kawasaki308 variant was identified in aerosols collected from a hospital setting [21], indicating its ongoing environmental circulation. Nevertheless, most studies of global norovirus gastroenteritis have focused on pediatric patients. The present study aimed to assess the prevalence and genotype of norovirus in clinical samples obtained from adult patients during 2021−2023. Molecular methods were employed to determine norovirus infection status, genotype classification based on capsid and polymerase sequences, and viral loads in stool samples as part of genomic surveillance.

## Materials and methods

### Ethics approval

This study was granted exemption by the Ethical Review Committee for Human Research, Faculty of Public Health, Mahidol University (Protocol No. 156/2564); the need for consent was waived. From 1 December 2021–31 March 2023, the stool samples from two hospitals were collected for routine investigation in microbiological laboratory. The present study obtained these samples from the laboratory and there was no direct interaction with patients. All data from medical records were anonymized prior to analysis to ensure patient confidentiality, in accordance with the Declaration of Helsinki.

### Stool samples

A hospital-based cross-sectional study was conducted during 2021–2023. A total of 250 stool samples were collected from patients of all ages with acute gastroenteritis who attended two hospitals—Uttaradit Hospital and Fort Pichaidabhak Hospital—in Uttaradit Province, located in the north of Thailand. These samples were sent to microbiological laboratory for routine investigation. Meanwhile, the samples were diluted at a ratio of 1:10 in phosphate-buffered saline (0.01 M PBS, pH 7.2–7.4) and tested for norovirus genogroups GI and GII.

### Nucleic acid extraction

A 200 µL of each diluted stool sample (1:10) was used for viral RNA extraction using the QIAamp^®^ Viral RNA Mini Kit (QIAGEN GmbH, Hidden, Germany), following the manufacturer’s protocol. The extracted RNA was eluted in 60 μL nuclease-free water and used for downstream amplification.

### RT-nested PCR

The presence of norovirus RNA in stool samples was determined using reverse transcription-nested polymerase chain reaction (RT-nested PCR), as described by Kittigul et al. [22]. One-step RT-PCR was performed separately for norovirus GI and GII in a 50 µL reaction volume using reagents from Invitrogen (Carlsbad, CA, USA). Each reaction contained 2 µL of denatured RNA and 48 µL of RT-PCR mixture consisting of 1X Reaction Mix (containing 0.2 mM each dNTP and 1.6 mM MgSO_4_), SuperScript^TM^ III RT/Platinum^®^ *Taq* Mix, 0.33 µM primers (COG1F and G1-SKR for GI; COG2F and G2-SKR for GII), and nuclease-free water. The RT-PCR cycling conditions included reverse transcription at 42 ºC for 60 min, initial denaturation at 94 ºC for 2 min, followed by 35 cycles at 94 ºC for 1 min, 50 ºC for 1 min, and 72 ºC for 1 min, with a final extension at 72 ºC for 3 min. Nested PCR was then carried out in a 50 µL reaction volume. Two microliters of the RT-PCR product were added to 48 µL of nested PCR mixture containing of 1X PCR buffer, 2.5 mM MgCl_2_, 0.2 mM each dNTP, 2.5 U for *Taq* DNA polymerase (Invitrogen, Carlsbad, CA), 0.33 µM nested primers (G1-SKF and G1-SKR for GI; G2-SKF and G2-SKR for GII), and nuclease-free water. The PCR conditions were as follows: initial denaturation at 94 ºC for 3 min, followed by 35 cycles for GI or 30 cycles for GII of 94 ºC for 1 min, 50 ºC for 1 min, and 72 ºC for 2 min, with a final extension at 72 ºC for 15 min. PCR products were analyzed by 1.5% agarose gel electrophoresis. Expected band sizes were 330 bp for norovirus GI and 344 bp for norovirus GII (S1 Fig).

### Polymerase-capsid typing assay

Norovirus GII-positive stool samples were further analyzed using a polymerase-capsid (PC) typing assay to determine the genotype and P-type, as described by Chhabra et al. [23], with some modifications. In the first round of the assay, RT-PCR was performed in a 50 µL reaction volume containing 5 µL denatured RNA and 45 µL of RT-PCR mixture. The mixture included 1X Reaction Mix, SuperScript^™^ III RT/ Platinum^®^*Taq* Mix, 0.33 µM of each primer (Mon431 and G2-SKR), and nuclease-free water. The cycling conditions were as follows: reverse transcription at 42 ºC for 30 min, initial denaturation at 95 ºC for 15 min, followed by 40 cycles of PCR at 95 ºC, 50 ºC, and 72 ºC for 1 min each, and a final extension at 72 ºC for 10 min. The second round of the assay was performed by adding 2 µL of the first round PCR product to 48 µL of the reaction mixture and amplifying under the same conditions as the first round. The expected PCR product size for norovirus GII was approximately 570 bp (S2 Fig).

### Semi-nested RT-PCR

Norovirus GII-positive stool samples were further analyzed using semi-nested RT-PCR with specific primer pairs targeting the RdRp and capsid genes (region B–C) to detect recombinant norovirus strains [22]. The first-round RT-PCR was performed using SuperScript^TM^III RT/Platinum^®^*Taq* Mix with GII primers JV12Y and G2SKR. Reaction conditions included reverse transcription at 42 °C for 60 min, initial denaturation at 94 °C for 2 min, followed by 45 cycles of amplification at 94 °C for 1 min, 50 °C for 1 min, and 72 °C for 2 min, with a final extension at 72 °C for 10 min. The second round of PCR was conducted using PCR Master Mix and GII primers p289IUB and G2SKR, under the same cycling conditions as the first round. The expected amplicon size for norovirus GII was approximately 1095 bp (S3 Fig).

### Norovirus genotyping and phylogenetic analysis

PCR products from norovirus GII-positive stool samples—detected by RT-nested PCR, PC typing, and semi-nested RT-PCR—were subjected to DNA sequencing**.** The resulting nucleotide sequences were aligned with reference norovirus strains from the NCBI GenBank database using the BLAST server. Genotypes and P-types were determined using the Norovirus Genotyping Tool (http://www.rivm.nl/mpf/norovirus/typingtool, RIVM, MA Bilthoven, Netherlands). Phylogenetic analysis was performed for partial capsid and polymerase gene sequences using MEGA 11 software to assess the genetic relationship between the norovirus strains identified in this study and global norovirus strains (S4 Fig).

### RT-qPCR

Extracted RNA from norovirus GII-positive stool samples was quantified to determine viral load using quantitative reverse transcription-polymerase chain reaction (RT‐qPCR) as previously described [22]. The one-step RT-qPCR was performed in a 20 μL reaction volume using the LightCycler RNA Master Hybprobe kit with *Tth* DNA polymerase (Roche Diagnostics, Mannheim, Germany). Briefly, 5 μL of RNA extract was added to 15 μL of reaction mixture containing 1X LightCycler RNA Master Hybprobe, *Tth* DNA polymerase buffer, dNTP mix, 3.25 mM Mn (OAc)_2_, 0.5 µM of primer QNIF2, 0.9 µM of primer COG2R, 0.25 µM of probe QNIFs, and nuclease-free water. Thermal cycling was carried out on the LightCycler 96 Real-Time PCR System (Roche Diagnostics) under the following conditions: reverse transcription at 55 °C for 30 min, enzyme activation at 95 °C for 5 min, followed by 45 amplification cycles of 95 °C for 15 s and 60 °C for 1 min. A standard curve was generated using 10-fold serial dilutions of norovirus GII RNA transcripts ranging from 1 × 10^3^ to 1 × 10^7^ RNA copies/mL. Cq values obtained from test samples were plotted against the standard curve to quantify genome copy numbers. Viral concentrations were calculated as genome copies per milliliter and converted to genome copies per gram of stool to determine the final viral loads.

### Statistical analysis

Statistical analyses were performed using IBM^®^ SPSS^®^ Statistics version 29. Associations between patient factors and norovirus infection were assessed using Fisher’s Exact test, except for gender, which was analyzed using the Chi-squared test. Comparisons of viral loads among different norovirus genotypes were performed using one-way ANOVA with post hoc analysis. *P-*values ≤ 0.05 were considered statistically significant.

## Results

### Prevalence of norovirus infection

A total of 250 stool samples were collected from patients with acute gastroenteritis aged 12–84 years and tested for noroviruses GI and GII using RT-nested PCR. All samples tested negative for norovirus GI. Norovirus GII was identified in 28 samples (11.2%) by DNA sequencing. Norovirus GII infection was detected in patients aged 22–72 years. Nine patients aged 12–20 years tested negative for norovirus RNA and were excluded from the age group analysis. Based on the proportion of infected patients within each age group, the highest infection rate was observed in individuals aged ≥66 years, followed by the 36–50, 21–35, and 51–65-year age groups. However, the differences were not statistically significant (*p* = 0.136). Females had a higher rate of norovirus GII infection than males, though the difference was not statistically significant (*p* = 0.227). All infected patients were outpatients and appeared to have a higher infection rate than inpatients, but the difference was not significant (*p* = 0.372) (Table 1).

**Table 1:** Characteristics of patients infected with norovirus GII.

Patient characteristics^a^	No. norovirus GII/Total GII-positive samples (%)	No. norovirus GII/Total samples in each group (%)	P-value
**Age, years**			0.136^b^
21–35	4/28 (14.3)	4/52 (7.7)	
36–50	15/28 (53.6)	15/94 (16.0)	
51–65	4/28 (14.3)	4/68 (5.9)	
≥66	5/28 (17.8)	5/27 (18.5)	
**Gender**			
Male	9/28 (32.1)	9/110 (8.2)	0.227^c^
Female	19/28 (67.9)	19/140 (13.6)	
**Patients attending**			
OPD	28/28 (100.0)	28/237 (11.8)	0.372^b^
IPD	0/28 (0.0)	0/13 (0.0)	

^a^A total of 241 cases was analyzed by age group, while 250 cases were included for gender, and OPD/IPD analysis. OPD, outpatient department. IPD, inpatient department.

^b^Fisher’s Exact test.

^c^Chi-squared test.

Over the 16-month study period (December 2021–March 2023), norovirus GII was detected in stool samples collected during several months: December 2021; February to July 2022, December 2022; and January to March 2023. The highest detection rate occurred in March 2023 (44.4%), followed by December 2021 (28.6%), March 2022 (25.0%), and January and February 2023 (20.0% each).

### Norovirus genotypes and P-types

**Fig 1:**
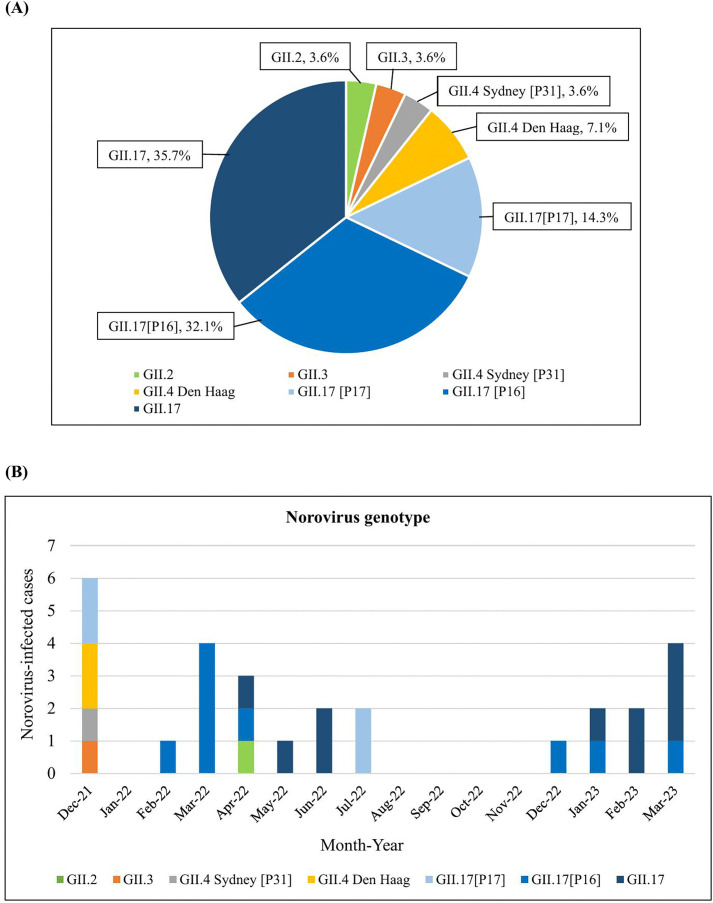
Distribution of norovirus genotypes and P-types detected in patients with acute gastroenteritis attending two hospitals in Thailand. **(A)** Detection rate of norovirus GII. **(B)** Monthly distribution of norovirus GII from December 2021 to March 2023.

Among the 28 norovirus GII-positive samples, the most frequent detected norovirus genotype based on capsid sequence was GII.17 (82.1%), followed by GII.4 (10.7%), GII.3 (3.6%), and GII.2 (3.6%). The 23 GII.17 sequences showed 98–100% nucleotide identity and were closely related to the reference strains: Kawasaki308/JPN/2015 (LC037415) and Kawasaki323/JPN/2014 (AB983218). These strains were similar to the GII.17 strains detected between 2014 and 2019 in Japan, Korea, China, Brazil, and the USA. Fourteen (50.0%) samples could be genotyped using the PC typing assay. Regarding capsid (VP1) and polymerase (RdRp) sequences, GII.17[P16] was the predominant strain (9 samples, 64.3%), followed by GII.17[P17] (2 samples, 14.3%), GII.17[PNA] (2 samples, 14.3%), and GII.4 Sydney 2012[PNA] (1 sample, 7.1%). The notation [PNA] indicates P-types that could not be assigned. Twelve (42.9%) samples were genotyped using the semi-nested RT-PCR. The identified strains included recombinant genotypes GII.17[P16] (7 samples, 58.3%) and GII.4 Sydney 2012[P31] (1 sample, 8.3%), as well as non-recombinant GII.17[P17] (4 samples, 33.3%). The GII.17[P17] strains were closely related to the Kawasaki308/JPN/2015 strain. The detected GII.P16 strains were similar to the GII.P16 derived from recombinant GII.4 Sydney[P16] strains which were found in Canada in 2023 and Thailand in 2023. Of note, two GII.17[P17] and one GII.4 Sydney 2012[P31] samples that were unassigned by PC typing could be successfully typed by semi-nested RT-PCR. Two samples identified as GII.17[P16] by PC typing were negative by semi-nested RT-PCR. Overall, GII.17 was the most prevalent genotype, frequently detected in combination with both GII.P16 and GII.P17. The most common genotype was GII.17 (10/28, 35.7%), followed by GII.17[P16] (9/28, 32.1%) and GII.17[P17] (4/28, 14.3%). GII.4 Den Haag (2/28, 7.1%), and GII.4 Sydney 2012[P31], GII.3 and GII.2 (1/28, 3.6% each) were detected less frequently, as shown in Fig 1A. During 2021–2023, more than two different norovirus genotypes were found in December 2021 and April 2022. In most months, one or two genotypes were detected, with GII.17 appearing consistently, often in combination with GII.P16, GII.P17, or an unassigned P-type throughout the study period. Monthly and yearly distributions of norovirus genotypes are shown in Fig 1B.

### Viral loads in norovirus GII-infected cases

**Fig 2:**
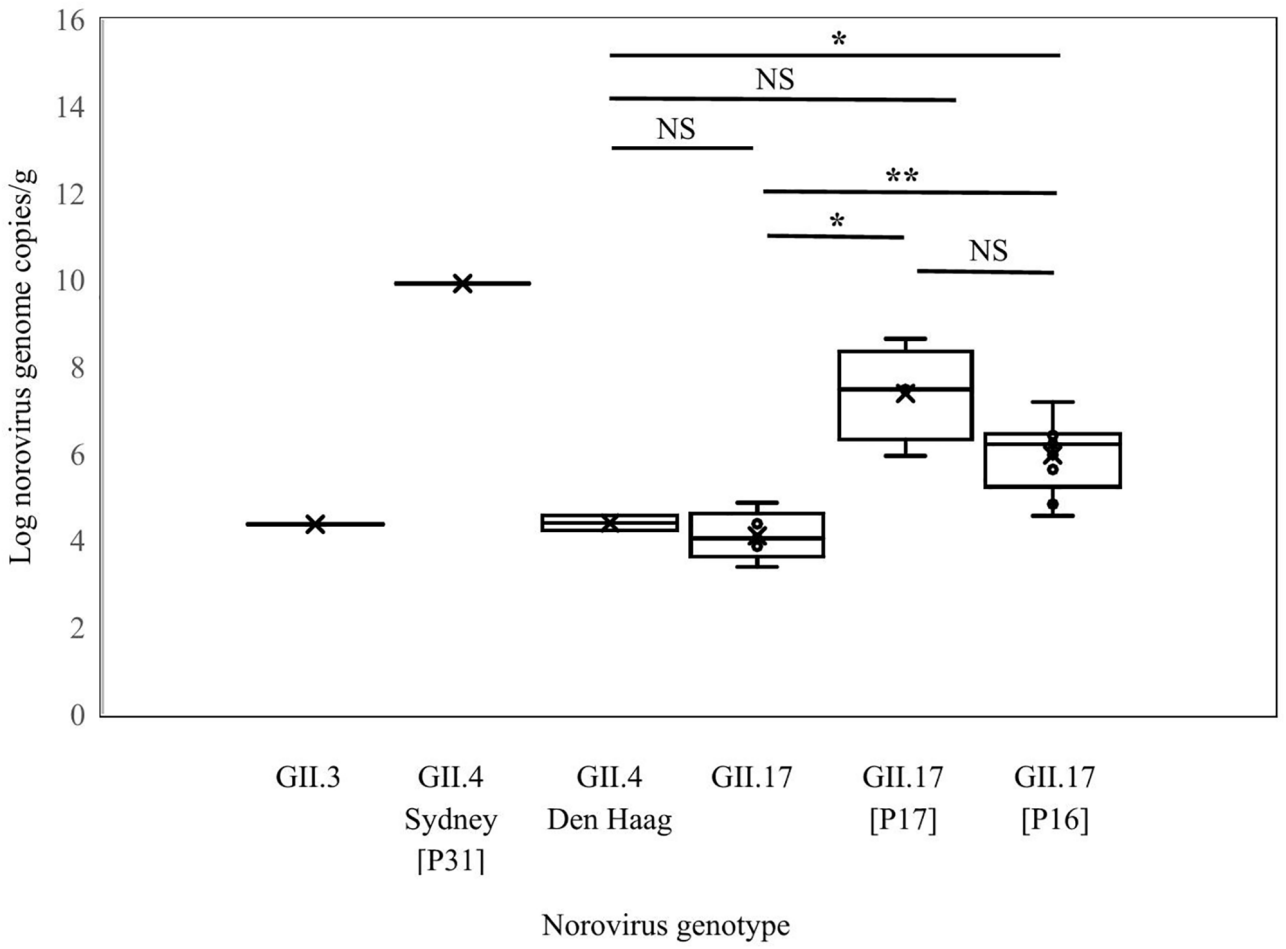
Difference in viral loads in stool samples from norovirus GII-infected patients, grouped by genotype. Bar graphs represent the mean ± standard deviation (S.D.) of viral loads from norovirus GII-positive stool samples. Statistical analysis was performed using one-way ANOVA by post hoc tests. *P* values ≤ 0.05 were considered statistically significant (*P < 0.05, **P < 0.01, ns = not significant).

Using RT-qPCR, norovirus GII RNA was detected in stool samples from 22 (78.6%) of the 28 norovirus GII-positive samples identified by RT-nested PCR, as shown in Fig 2. Quantification of norovirus genome copies showed values of 2.4 × 10^4^ in GII.3 (1 sample), 8.3 × 10^9^ in GII.4 Sydney 2012[P31] (1 sample), 1.7 × 10^4^ and 3.8 × 10^4^ in GII.4 Den Haag (2 samples), 2.4 × 10^3^–7.4 × 10^4^ in GII.17 (5 samples), 8.9 × 10^5^–4.5 × 10^8^ in GII.17[P17] (4 samples), and 3.7 × 10^4^–1.6 × 10^7^ genome copies/ g of stool in GII.17[P16]-infected cases (9 samples). The highest viral load was found in the patient infected with the epidemic recombinant strain GII.4 Sydney 2012[P31], while the lowest viral loads were observed in cases infected with GII.17 strains that could not be assigned a P-type. Comparison of norovirus concentrations and genotypes revealed statistically significant differences of viral load in GII.17[P17] and GII.17 (*p* = 0.029); GII.17[P16] and GII.4 Den Haag (*p* = 0.013); and GII.17[P16] and GII.17-infected cases (*p* = 0.002), as shown in Fig 2.

## Discussion

The genetic diversity and continual evolution of norovirus have led to shifts in the predominant genotypes circulating in human populations over time. This hospital-based study showed the prevalence of norovirus infection (11.2%) in adults consistent with a previous study conducted across all age groups (10.9%) [17]. In Thailand, norovirus prevalence in children under five years of age with acute gastroenteritis increased from 14.9% in 2017–2018 [18] to 18.7% in 2019–2020 [19]. The global pooled prevalence of norovirus gastroenteritis between 2012 and 2022 was 19.0%, with substantial variation across countries and age groups. Norovirus GII was consistently more prevalent than norovirus GI among all age groups [24]. In our study during 2021–2023, a lower prevalence was observed, and all positive cases were infected with norovirus GII. During the COVID-19 pandemic, children with acute gastroenteritis were more likely to be treated at home, while adults with diarrhea were more frequently hospitalized, likely due to heightened public health control measures. The age group ≥66 years appeared particularly vulnerable to norovirus infection. A previous report highlighted that the highest incidence of norovirus gastroenteritis occurred in children under five years of age, while the highest case fatality rate was observed in individuals aged 65 years and older [25]. Severe gastroenteritis due to norovirus was more common in both young children and the elderly [26]. In the present study, all norovirus-infected patients were outpatients exhibiting mild to moderate symptoms of acute gastroenteritis. There was no significant difference in infection rates between males and females, and no clear seasonality was observed—both findings consistent with previous research [19].

This study employed a combination of molecular techniques to detect norovirus GII RNA, determine its genotype and P-type, and quantify viral load in sporadic adult cases. Several genotypes were identified over the study period, with GII.17 emerging as the predominant strain, including GII.17[P16], GII.17[P17], and GII.17 strains with unidentified P-type. Other detected genotypes included GII.4 (specifically GII.4 Sydney 2012[P31] and GII.4 Den Haag), GII.3, and GII.2. In 2021, GII.2, GII.4, and GII.17 noroviruses were present, while nearly all norovirus infections in 2022 and 2023 were caused by GII.17, indicating sustained circulation of this genotype. The predominance of GII.17 aligns with findings from a study in China, which reported that GII.4, GII.3, and GII.2 strains were mainly detected in children, whereas GII.17 was more commonly found in adults with acute gastroenteritis [27].

Between 2021 and 2023, the detection of GII.17 and GII.17[P17] genotypes in this study was consistent with reports of norovirus outbreaks in Spain [12], China [14], and Romania [15]. In 2024, GII.17[P17] was identified as the causative agent of acute gastroenteritis outbreaks in Argentina [13], suggesting the continued global spread of this genotype over the past decade. However, GII.17[P17] strains detected in our study, with nucleotide identity closely related to Kawasaki308/2015 strain representing cluster D based on phylogenetic analysis [28] may differ from a recently new cluster of GII.17[P17] represented by Romania/2021 identified in the United States, England, Germany, France, and Austria during 2021−2024 [6]. To analyse evolutionary perspective, further studies on DNA sequencing of full-length VP1 and RdRp sequences are needed to provide more detailed genetic characterization of GII.17[P17] strains. The GII.17 strains found in our study were consistent with previous findings from oysters, recycled water, sewage sludge, and air samples in Thailand [21,22,28] exhibiting their circulation in human and environment. Notably, both GII.17[P17] and GII.17[P16] were detected, with GII.17[P16] being the most frequently observed. These findings may reflect an ongoing epidemiological shift in norovirus genotypes across different geographic regions.

Recombinant GII.17 viruses were previously detected with GII.P3, GII.P4, GII.P13, GII.P16, GII.P25, and GII.P38 in patients with acute gastroenteritis [9,29]. In this study, the predominant GII.17 capsid harbored the GII.P16 polymerase type. GII.17[P16] strains emerged in 2022 and persisted through 2023 alongside GII.17[P17]. To our knowledge, this is the first report documenting the emergence and predominance of GII.17[P16] strains over the past decade. GII.17[P16] was previously detected in patients with acute gastroenteritis in South Africa, 2011 [9] and United Kingdom, 2014 [30]. Interestingly, the present study identified the emergence of recombinant GII.17[P16] norovirus strains, supporting their ability to switch RdRp sequences through recombination. The ability of GII.17 viruses to exchange RdRp sequences and generate new recombinant strains may contribute to their persistence in the population, enhanced transmissibility, and ongoing molecular evolution. Recombinant norovirus strains carrying the GII.P16 polymerase have notably increased and caused acute gastroenteritis illness outbreaks, including GII.4 Sydney[P16] and GII.2[P16], as reported in Brazil [8], Australia and New Zealand [31], China [32], and Japan [33]. These findings suggest that the polymerase GII.P16, rather than the VP1 capsid, may play a key role in the outbreak potential of norovirus gastroenteritis [32].

Regarding norovirus shedding, although the recombinant GII.4 Sydney 2012[P31] was found in only one sample, it exhibited the highest concentration (approximately 10^9^ genome copies/g of stool), which aligns with previous findings in China where GII.4 Sydney strains had the highest viral loads [34]. This well-known outbreak strain may contribute to rapid community transmission. Stool samples with viral loads around 10^7^ genome copies/g were also consistent with observations in Brazil [8]. The association between viral loads and norovirus genotype demonstrated that GII.17[P17] (10^5^–10^8^ genome copies/g) had a significantly higher viral load than non-typed GII.17 strains (10^3^–10^4^ genome copies/g). Similarly, GII.17[P16] (10^4^–10^7^ genome copies/g) showed significantly higher viral loads than both GII.17 and GII.4 Den Haag strains (10^4^ genome copies/g). Notably, low viral shedding levels (approximately 10^3^–10^4^ genome copies/g) were observed in samples where P-type could not be determined, such as GII.3, GII.4 Den Haag, and GII.17 strains. It is likely that the GII.2 strain, for which viral load could not be determined, also carried a lower concentration (<10^3^ genome copies/g) in stool. A previous study in Hong Kong (2012–2017) reported a higher viral load of emerging recombinant norovirus GII.2[P16] than GII.4[Pe] and GII.17[P17] among older children, adults, and the elderly. However, only threshold cycle (Ct) values were determined using RT-qPCR; the virus concentrations were not evaluated [35]. The discordant results may be due to the difference in the study year; GII.2[P16] emerged in 2016 [36], whereas after pandemic COVID-19, GII.17[P17] strains have been increasingly reported [6,12–16].

This study has limitations. First, only partial nucleotide sequences of norovirus were analyzed: whole genome sequencing of VP1 and RdRp sequences would provide complete information of genetic diversity. Second, small numbers of norovirus-positive samples could be determined for viral load which limited to address the trend of viral load by genotypes. Viral load in stool samples might be also varied by timing of stool collection, age of patients, signs and symptoms of patients with acute gastroenteritis.

The present study highlights the predominance of norovirus GII.17, particularly GII.17[P17] and recombinant GII.17[P16] strains, which were detected at significant viral loads and associated with gastrointestinal illness in adults. Given the genetic diversity and rapid evolution of norovirus, the predominant genotypes may change unpredictably. Further studies are needed to explore the potential role of emerging genotypes in norovirus outbreaks and to support vaccine development for effective prevention and control.

## Supporting information

S1 Fig Amplicons of norovirus GII in the stool samples detected using RT-nested PCR. (PDF)

S2 Fig Amplicons of norovirus GII in the stool samples detected using PC typing. (PDF)

S3 Fig Amplicons of norovirus GII in the stool samples detected using semi-nested RT-PCR. (PDF)

S4 Fig Phylogenetic analysis of partial norovirus GII nucleotide sequences in comparison with reference strains. (PDF)
