# Supplementary material for: Emergence of norovirus GII.17[P16] in adult patients with acute gastroenteritis in Thailand during 2021−2023
Source: PLoS One. 2025 Nov 24;20(11):e0337513. doi: 10.1371/journal.pone.0337513 (PMC12643282; doi:10.1371/journal.pone.0337513)
Supplement: S1 Fig — (PDF) [file pone.0337513.s001.pdf]

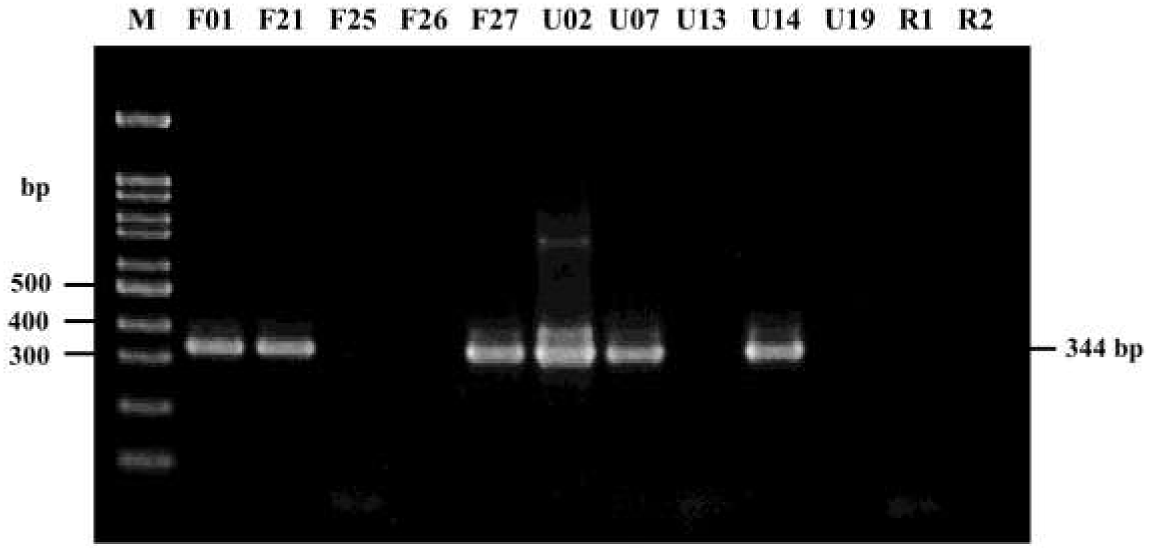

S1 Fig. Amplicons of norovirus GII in the stool samples detected using RT-nested PCR. Lane: M, DNA marker (100 bp DNA Ladder); F01–F27, and U02–U19: the stool samples from two hospitals; R1 and R2, negative controls of the first round PCR and the second round PCR, respectively. Gel electrophoresis of the RT-nested PCR products of norovirus GII showed 344 bp in lanes F01, F21, F27, U02, U07, and U14.
