# Supplementary material for: Emergence of norovirus GII.17[P16] in adult patients with acute gastroenteritis in Thailand during 2021−2023
Source: PLoS One. 2025 Nov 24;20(11):e0337513. doi: 10.1371/journal.pone.0337513 (PMC12643282; doi:10.1371/journal.pone.0337513)
Supplement: S2 Fig — (PDF) [file pone.0337513.s002.pdf]

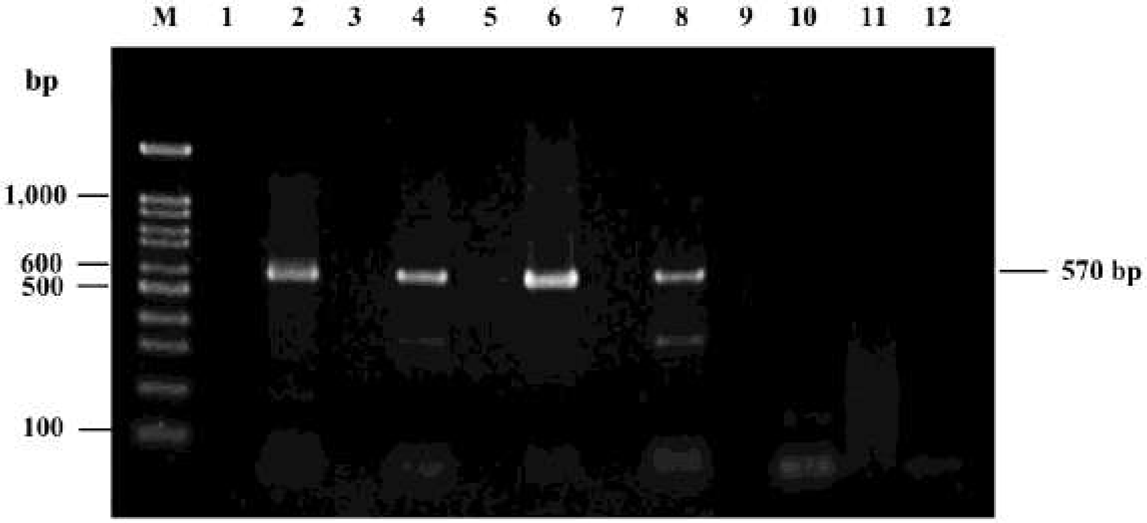

S2 Fig. Amplicons of norovirus GII in the stool samples detected using PC typing. Each sample was amplified by the first round PC assay (RT-PCR) and the second round PC assay (nested PCR). Lane: M, DNA marker (100 bp DNA Ladder); 1, 3, 5, 7 and 9, RT-PCR products from stool samples; 2, 4, 6, 8 and 10, secondary PCR products from the corresponding stool samples; 11, negative control of RT-PCR; and 12, negative control of nested PCR. Gel electrophoresis of the nested PCR products of norovirus GII showed 570 bp in lanes 2, 4, 6, and 8.
