# Supplementary material for: Emergence of norovirus GII.17[P16] in adult patients with acute gastroenteritis in Thailand during 2021−2023
Source: PLoS One. 2025 Nov 24;20(11):e0337513. doi: 10.1371/journal.pone.0337513 (PMC12643282; doi:10.1371/journal.pone.0337513)
Supplement: S3 Fig — (PDF) [file pone.0337513.s003.pdf]

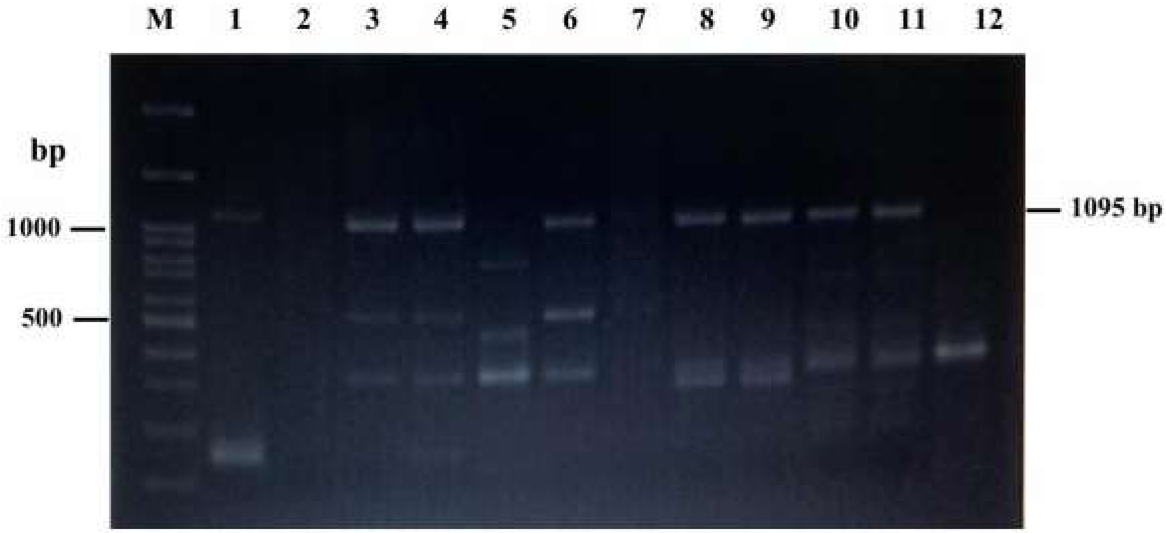

S3 Fig. Amplicons of norovirus GII in the stool samples detected using semi-nested RT-PCR. Lane: M, DNA marker (100 bp DNA Ladder); 1–12 PCR products from stool samples. Gel electrophoresis of the nested PCR products of norovirus GII showed 1095 bp in lanes 3, 4, 6, and 8–11.
