# Supplementary material for: Emergence of norovirus GII.17[P16] in adult patients with acute gastroenteritis in Thailand during 2021−2023
Source: PLoS One. 2025 Nov 24;20(11):e0337513. doi: 10.1371/journal.pone.0337513 (PMC12643282; doi:10.1371/journal.pone.0337513)
Supplement: S4 Fig — (PDF) [file pone.0337513.s004.pdf]

(A)

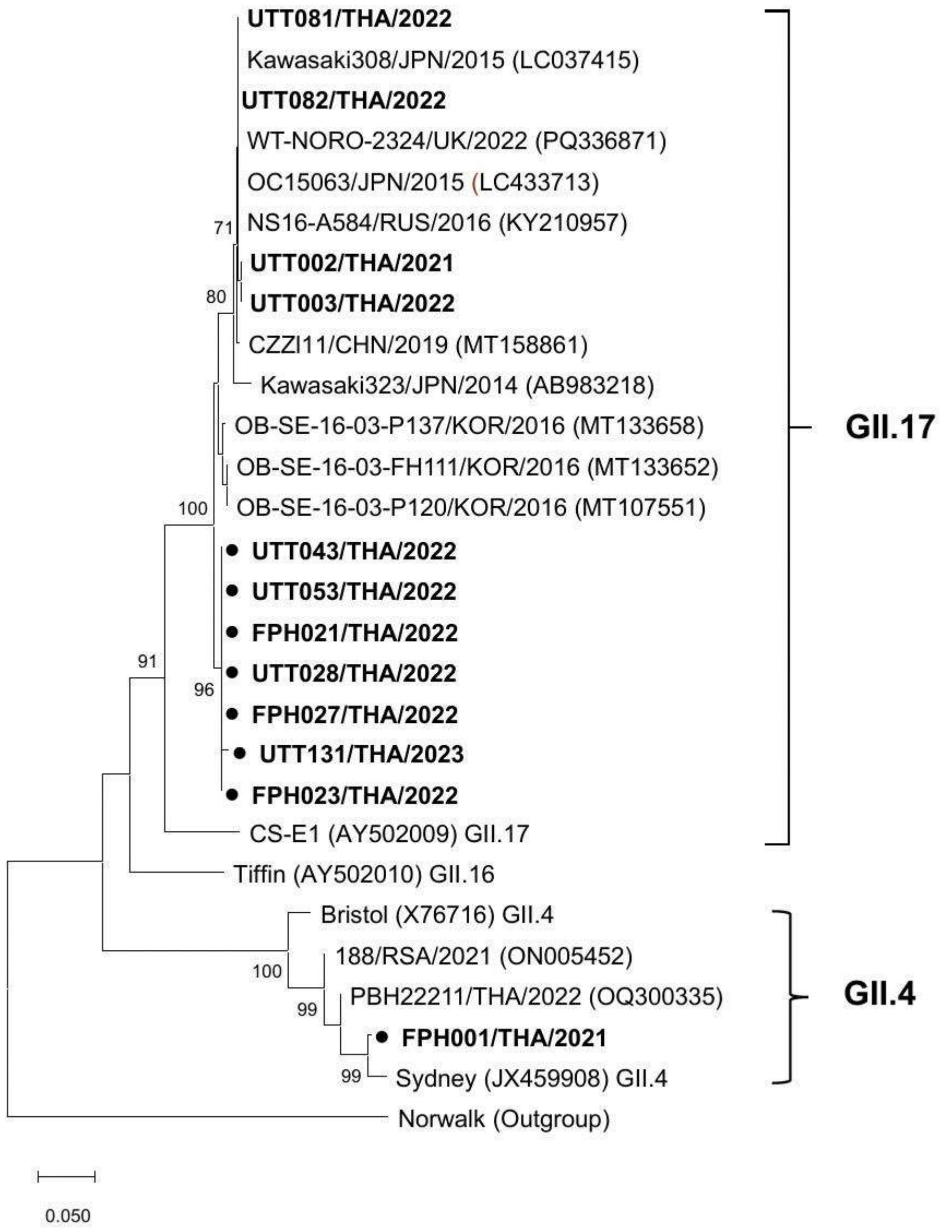
UTT081/THA/2022
Kawasaki308/JPN/2015 (LC037415)
UTT082/THA/2022
WT-NORO-2324/UK/2022 (PQ336871)
OC15063/JPN/2015 (LC433713)
NS16-A584/RUS/2016 (KY210957)
71
UTT002/THA/2021
80
UTT003/THA/2022
CZZI11/CHN/2019 (MT158861)
Kawasaki323/JPN/2014 (AB983218)
OB-SE-16-03-P137/KOR/2016 (MT133658)
OB-SE-16-03-FH111/KOR/2016 (MT133652)
100
OB-SE-16-03-P120/KOR/2016 (MT107551)
UTT043/THA/2022
UTT053/THA/2022
FPH021/THA/2022
91
UTT028/THA/2022
96
FPH027/THA/2022
UTT131/THA/2023
FPH023/THA/2022
CS-E1 (AY502009) GII.17
Tiffin (AY502010) GII.16
Bristol (X76716) GII.4
188/RSA/2021 (ON005452)
100
PBH22211/THA/2022 (OQ300335)
99
FPH001/THA/2021
99
Sydney (JX459908) GII.4
Norwalk (Outgroup)
GII.17
GII.4
0.050

(B)

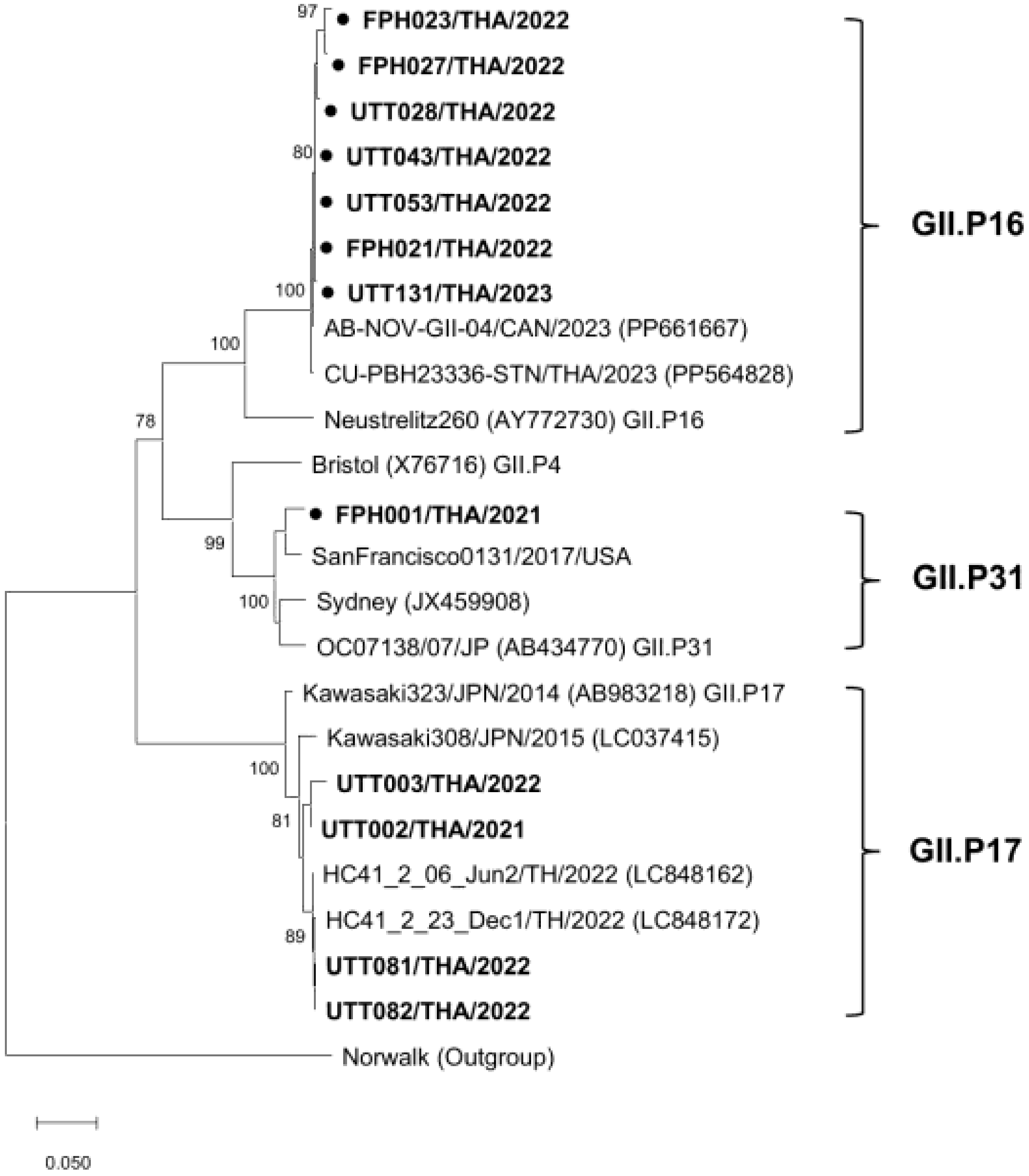
FPH023/THA/2022
FPH027/THA/2022
UTT028/THA/2022
UTT043/THA/2022
UTT053/THA/2022
FPH021/THA/2022
UTT131/THA/2023
AB-NOV-GII-04/CAN/2023 (PP661667)
CU-PBH23336-STN/THA/2023 (PP564828)
Neustrelitz260 (AY772730) GII.P16
Bristol (X76716) GII.P4
FPH001/THA/2021
SanFrancisco0131/2017/USA
Sydney (JX459908)
OC07138/07/JP (AB434770) GII.P31
Kawasaki323/JPN/2014 (AB983218) GII.P17
Kawasaki308/JPN/2015 (LC037415)
UTT003/THA/2022
UTT002/THA/2021
HC41_2_06_Jun2/TH/2022 (LC848162)
HC41_2_23_Dec1/TH/2022 (LC848172)
UTT081/THA/2022
UTT082/THA/2022
Norwalk (Outgroup)
GII.P16
GII.P31
GII.P17
97
80
100
100
78
99
100
100
81
89
0.050

S4 Fig. Phylogenetic analysis of partial norovirus GII nucleotide sequences in comparison with reference strains. (A) The VP1 capsid region. (B) The RdRp region. The phylogenetic trees of norovirus GII strains detected in this study using semi-nested RT-PCR (UTT002–UTT131 and FPH001–FPH028) and reference sequences were constructed using the neighbor-joining method. Evolutionary analyses were conducted in MEGA 11.0 with 1000 bootstrap replicates based on the Kimura two-parameter model. Bootstrap values greater than 70% are shown at the branch nodes. The scale bars represent nucleotide distances. Nucleotide sequences obtained in this study are shown in bold, and recombinant norovirus GII strains are marked with solid circles.
